# Family-Based Tag Rugby: Acute Effects on Risk Factors for Cardiometabolic Disease and Cognition and Factors Affecting Family Enjoyment and Feasibility

**DOI:** 10.3390/healthcare13243186

**Published:** 2025-12-05

**Authors:** Scarlett M. Fountain, Grace W. M. Walters, Ryan A. Williams, Caroline Sunderland, Simon B. Cooper, Karah J. Dring

**Affiliations:** Sport Health and Performance Enhancement (SHAPE) Research Centre, Department of Sport Science, School of Science & Technology, Nottingham Trent University, Nottingham NG11 8NS, UK; grace.walters@ntu.ac.uk (G.W.M.W.); ryan.williams@ntu.ac.uk (R.A.W.); caroline.sunderland@ntu.ac.uk (C.S.); simon.cooper@ntu.ac.uk (S.B.C.); karah.dring@ntu.ac.uk (K.J.D.)

**Keywords:** family-based physical activity, cardiometabolic health, cognitive function, socioeconomic status

## Abstract

**Highlights:**

**What are the main findings?**

- An acute bout of family tag rugby can improve postprandial insulin concentration in parents and cognitive function in both children and their parents.
- Families from a range of socioeconomic backgrounds deem family-based tag rugby an appropriate exercise modality, which can be adapted to overcome the barriers associated with the cost of and access to local facilities for low socioeconomic status families.

**What are the implications of the main findings?**

- Family-based tag rugby elicits certain health (parents only) and cognitive benefits (children and parents) whilst being an enjoyable form of activity with potential for long-term implementation.
- Family-based tag rugby may be an avenue for reducing the barriers that low socioec-onomic status families face with the cost of, access to, and provision of local physical activity facilities.

**Abstract:**

**Background/Objectives**: Physical inactivity is associated with increased cardiometabolic disease risk and poor cognition in children and their parents. Family-based physical activity offers an opportunity for children and their parents to engage in physical activity concurrently. The present study examined the effect of an acute bout of family-based tag rugby on risk factors for cardiometabolic disease and cognition in families. Additionally, this study qualitatively explored families’ perceptions of enjoyment and factors affecting implementation with considerations for socioeconomic status. **Methods**: Sixteen families (27 children, 20 parents) participated in an exercise (45 min family-based tag rugby) and resting control trial (45 min seated rest), separated by seven days. Postprandial gylcaemia, insulinaemia, lipaemia and cognitive function were measured following exercise/rest. Families also participated in whole-family focus groups and separate parent and child interviews. **Results**: In parents, postprandial plasma insulin concentrations were lower on the exercise trial than the rested control trial at 30 min (*p* = 0.004) and 120 min following the consumption of a standardised lunch (*p* = 0.011). In children, a significant trial*time interaction for inverse efficiency scores on the Sternberg paradigm (three-item) was exhibited (*p* = 0.016). In parents, a significant trial*time interaction for inverse efficiency score on the Stroop congruent test was exhibited (trial*time interaction; *p* = 0.012), whereby inverse efficiency scores improved immediately post-exercise, compared with the rested control trial (*p* = 0.016). Qualitatively, families from all socioeconomic backgrounds agreed that tag rugby is an inclusive, enjoyable mode of physical activity that families want to participate in together, which can be adapted to overcome the barriers associated with the cost of and access to local facilities. **Conclusions**: An acute bout of tag rugby improved postprandial insulin concentrations in parents and cognitive function in children and their parents. Tag rugby was deemed an appropriate exercise modality for families from a range of socioeconomic backgrounds.

## 1. Introduction

In the UK, 52% of children do not meet the physical activity guidelines of a minimum of 60 min of moderate-to-vigorous physical activity per day [1]. Specifically, children are less engaged with physical activity of all intensities during evenings and weekends when they are under the care of their parents [2,3]. Furthermore, parents are a known subgroup of the adult population who participate in less physical activity than their non-parent counterparts [4,5,6]. Physical inactivity in children and their parents is associated with increased risk factors for cardiometabolic diseases [7] and impaired cognitive function [8,9,10]. Thus, there is an urgent need for physical activity interventions that support children and their parents to become more physically active together during evenings and weekends when physical activity levels are lowest [2,3].

Most physical activity interventions are designed for children only and are typically delivered in a school setting [11,12,13], which does not address concerns relating to low physical activity levels during evenings and weekends [2,3], nor the low physical activity levels of parents [4]. Family-based physical activity provides an opportunity to engage the family unit with exercise during unstructured times [2,3]. Yet very little is known of the potential for family-based physical activity to enhance cardiometabolic health and cognitive function within the family unit. Many prior family-based physical activity interventions have focused on child physical activity levels only; for review, see Kriemler et al. [12]. Recently, a father and daughter physical activity intervention increased the father and daughter’s physical activity levels and reduced sedentary time [14]. Whilst promising, this is the first study, to the authors’ knowledge, that has examined co-participation in physical activity within the family unit, but has not examined whether these benefits extend to enhancing the cardiometabolic health or cognitive function of fathers and daughters. Furthermore, it is important that mothers are also involved in the co-participation physical activity opportunity, given their low physical activity levels [15].

Prior acute exercise interventions in children have reported that intermittent games-based activity effectively enhances cardiometabolic health (including anti-inflammatory cytokines and insulin sensitivity) and cognitive function [16,17,18]. Similar effects of acute bouts of intermittent games-based activity have been observed in adults for cardiometabolic health parameters [19,20] and cognition [21,22,23]. However, no study to date has explored the effect of an acute bout of family, games-based physical activity on cardiometabolic health and cognitive function in children and their parents. Examining the acute cardiometabolic and cognitive responses to family physical activity could help determine whether the chosen mode of physical activity enhances cardiometabolic health and cognition in families, to then be considered for the design of regular exercise interventions. In this instance, tag rugby was selected as the preferred activity because its non-contact format enables safe co-participation for children and parents, while its intermittent demands support anticipated metabolic responses and cognitively engaging, decision-intensive gameplay [16,17,18]. Moreover, prior family-based interventions have largely targeted children’s physical activity [12], with far less attention given to cardiometabolic and cognitive outcomes in either children or their parents. The present study sought to address this gap by promoting co-participation in tag rugby and assessing these broader health markers.

When designing family physical activity interventions, it is also important that the facilitators and barriers to engagement (in children and their parents) are considered to increase the likelihood of long-term adherence [24]. However, reflections on the accessibility and appeal of a selected mode of physical activity tend to occur after families have completed a chronic physical activity intervention [25,26,27], with no prior considerations made when the intervention is designed and developed. Such reflections following a single session, such as an acute bout of the chosen exercise modality, would provide important insight into the design of future family physical activity interventions to enhance the likelihood of success. Additionally, including families from various socioeconomic backgrounds is crucial to making the intervention accessible to all, and capturing family perceptions of enjoyment and feasibility helps tailor future interventions to meet the needs of families across different socioeconomic contexts [28].

Therefore, the aim of the present study was to examine the effect of an acute bout of family tag rugby on postprandial glycaemic, insulinaemic, and lipaemic responses, and cognitive function in children and their parents. A secondary aim of the study was to ascertain whether cardiometabolic and cognitive responses to the mode of activity exhibited similar trends across generations. A final aim of the study was to qualitatively explore families’ perceptions of their enjoyment of the family tag rugby session and factors affecting future implementation, with considerations for socioeconomic status.

## 2. Materials and Methods

### 2.1. Participant Characteristics

Forty-seven participants (16 families; 27 children, 20 parents) participated in the study (see Table 1 for the number of families, children, parents, and respective socioeconomic status group). The a priori sample size calculation reported in Section 2.7 indicated that 24 parents were required; however, only 20 were recruited. This shortfall may have reduced the statistical power of the study. Body mass was measured to the nearest 0.1 kg using a Seca 770 digital scale (Seca, Hamburg, Germany), and stature and sitting stature (children only) were measured to the nearest 0.1 cm using a Leicester Height Measure (Seca, Hamburg, Germany). Body mass and stature were used to calculate body mass index (BMI) for children and parents, and BMI percentiles for children only (BMI and BMI percentiles were utilised for descriptive purposes only). Participant leg length (children only) was calculated using sitting stature and stretched stature (leg length = stretched height − sitting height), to predict maturity offset [29]. Table 2 presents the descriptive characteristics of the participants.

### 2.2. Study Design

The study was approved by the institution’s ethical advisory committee (application no. 1557662) and employed a randomised, counterbalanced, crossover, within-subjects design. Due to the difficulties in recruiting families, randomisation was implemented at the family level using a non-stratified sequential enrolment approach. Written informed consent, parental consent, as well as child assent were obtained. The following exclusion criteria were applied: families without a child aged 8–16 years, any participant with a relative who has died during or soon after exercise following heart complications, and any factor that would cause an inability to complete the exercise component of the study. A parent/guardian completed health screen questionnaires on behalf of themselves and their child to ensure there were no medical conditions affecting participation in the study.

Participants completed a familiarisation session and two main trials, with all visits separated by ~7 d. Familiarisation consisted of demographic and lifestyle questionnaires, anthropometrics, a practice of the cognitive function tests, and a brief overview of the exercise and resting trials. Participants were asked to refrain from physical activity 24 h prior to the main trials and to arrive fasted from ~9 pm the previous night; diet was matched for 24 h prior to the main trials via participants recording their dietary intake. On arrival at the main trials, participants were fitted with a heart rate monitor (Team Sport System, Firstbeat Technologies Ltd., Jyvaskyla, Finland).

### 2.3. Socioeconomic Status Classification

The UK IMD score 2019 [30] was used to measure the residential area of deprivation and used as a marker of socioeconomic status. Families were ranked according to their corresponding IMD score, and the ranked data were organised into deciles, decile 1 being the most deprived and 10 being the least deprived. Families were organised into three groups based on their IMD score. Families in the ‘low socioeconomic status’ group were from deciles 1–3, families in the ‘middle socioeconomic status’ group were from deciles 4–7, and families in the ‘high socioeconomic status’ group were from deciles 8–10.

### 2.4. Experimental Procedures

#### 2.4.1. Standardised Breakfast and Lunch

A standardised breakfast and lunch were provided for all participants during the main trials. The breakfast consisted of cornflakes, milk, toast, and margarine, providing 1.5 g of carbohydrates per kg of body mass. Lunch consisted of a chicken or cheese sandwich (vegetarian option), baked crisps, and an apple, which also provided 1.5 g of carbohydrates per kg of body mass. The standardised lunch was provided 1-h post-exercise/rest. Both meals have been utilised in the previous literature examining postprandial responses to acute bouts of exercise [16,17,18].

#### 2.4.2. Capillary Blood Samples

To support compliance with fasting, the lead researcher sent reminder messages on the day of the evening fast. Fasting adherence was then verbally confirmed with participants upon arrival, prior to blood sample collection. Main trials involved collecting capillary blood samples in a fasted and rested state, followed by samples taken immediately after exercise or rest, and again 60 min later. To assess the postprandial responses, further samples were collected at 30, 60, and 120 minutes after lunch. Prior to each collection, participants’ hands were submerged in warm water for 5 min to increase capillary blood flow and provide a mild numbing sensation. For child participants, additional measures were taken to ensure comfort during blood sampling, including allowing time to familiarise themselves with the procedure in a calm and supportive environment. A single-use lancet (Unistik Extra, 21 gauge, 2.0 mm depth; Owen Mumford Ltd., Oxford, UK) was used to obtain each sample. Blood was collected into two 300 μL EDTA-coated microvettes for all samples (Sarstedt Ltd., Nümbrecht, Germany). A single 25 μL whole blood sample was also collected at all time points, using a pre-calibrated glass pipette (Hawksley Ltd., Brighton, UK), and immediately deproteinised in 250 μL ice-cooled 2.5% perchloric acid. Samples were centrifuged at 1000× *g* for 4 min at 4 °C (Eppendorf 5415 C, Hamburg, Germany). Plasma was removed from the microvettes and placed into 250 μL plastic vials for subsequent analysis. All samples were frozen immediately at −80 °C.

Blood glucose concentrations were determined in duplicate, using a commercially available assay (GOD/PAP method, GL364, Randox, Crumlin, Ireland). Plasma insulin concentrations were determined using a commercially available ELISA (Mercodia Ltd., Uppsala, Sweden). Plasma triglyceride concentrations were determined in a single sample utilising the GPO-PAP method (Randox, Ireland). The coefficient of variation, based on eight repeat measurements, was 3.2% for blood glucose concentration, 10% for plasma insulin concentration, and 3.1% for plasma triglyceride concentration. Glucose, insulin, and triglyceride incremental area under the curve (iAUC) following the standardised lunch were calculated utilising the trapezoid method described by Wolever and Jenkins [31]. Blood glucose, plasma insulin, and plasma triglyceride peak values were calculated as the individual peak for each participant following the standardised lunch.

#### 2.4.3. Cognitive Function Tests

Cognitive function tests were completed during both main trials. Tests were completed 30 min pre-, immediately post, and 45 min post-exercise or rest. The series of cognitive function tests lasted ~15 min and consisted of the Stroop test (a measure of information processing and inhibitory control), Sternberg paradigm (a measure of working memory) and a Flanker task (a measure of information processing and inhibitory control), and were completed on a laptop (Microsoft Surface Laptop Go 3; Microsoft, Redmond, Washington, DC, USA). For each test and test level, inverse efficiency scores were calculated from the response times and accuracy. Inverse efficiency scores were utilised as the preferred metric of performance because they combine response times and accuracy into a single measure, thereby mitigating the influence of the speed–accuracy trade-off that can confound interpretation when either metric is considered alone [32].

#### 2.4.4. Exercise

Children and their parents participated in a mid-morning bout of family, games-based exercise (45 min), which was compared against the rested control trial (seated rest). The acute bout of exercise included an intermittent tag rugby session, which children and their parents completed as a family. The tag rugby session commenced 1 h after breakfast. The lead researcher delivered the sessions to families, with total group sizes of no more than 15 participants, on a university multi-use games area, or similar available facilities at local leisure centres. The tag rugby session consisted of a warm-up (5 min of jogging on the court followed by whole body, dynamic stretches), skill-based drills (including 15 min of passing, manoeuvring, and tactical drills at moderate intensity with planned progression in drill complexity and movement demands to build towards the small-sided games), and small-sided games (20 min performed at moderate–vigorous intensity). The session finished with a 5 min cool-down. Session content and timings were standardised to ensure consistent delivery across families. Participants were fitted with heart rate monitors (First Beat Technologies Ltd., Jyväskylä, Finland) at the start of the main trials, and the heart rate system was lapped during the 45 min of exercise to determine average and maximum heart rate as an indicator of exercise intensity. Maximum heart rate was calculated (220-age) for each participant to enable calculation of relative exercise intensities. Participants were also fitted with a GPS (Playertek, Louth, Ireland) during the 45 min of exercise to determine the distance (m) covered.

### 2.5. Focus Groups and Interviews

Whole-family focus groups and separate parent and child interviews were utilised to gauge the perceived enjoyment of the session, and the feasibility of implementing family-based tag rugby at home. The qualitative findings were used to provide additional insights into families’ experiences and perceptions of the exercise session, and to contexutalise and interpret the quantitative results where relevant. Nevertheless, the lack of formal triangulation means that the qualitative and quantitative data provide complementary rather than integrated evidence, which limits the extent to which causal inferences or fully integrated interpretations can be made. Focus groups and interviews were conducted during the last 60 min of the exercise trial, prior to the collection of the final capillary blood sample. All sessions took place in a private, enclosed room with only two researchers present to minimise external interference. The lead researcher followed a structured interview guide, encouraged open discussion, and assured participants that all views (positive or negative) were welcome to mitigate bias associated with leading the exercise session, focus groups, and interviews. For example, neutral, non-directive prompts were used to avoid steering participants’ responses, standardised follow-up questions were applied across all focus groups and interviews, and participants were reminded that their feedback would not affect their involvement in the study. A total of 6 families were in the low socioeconomic group, 5 families in the middle socioeconomic status group, and 5 in the high socioeconomic status group (see Table 1).

#### 2.5.1. Whole-Family Focus Groups

Focus groups consisted of five or six questions, all with relevant probes. An opening question asked participants whether they took part in physical activity, including the mode of physical activity, the frequency, and what could help them to participate if they are currently physically inactive. The questions then moved into details relating to their enjoyment and the feasibility of the family tag rugby session, and lasted between 10 and 20 min.

#### 2.5.2. Parent Interviews

Parent interviews were conducted based on family size, and therefore, if one parent was present, these were conducted as a single-parent interview, and if two parents were present, these were conducted as dyadic interviews. An opening question asked if parents preferred to take part in physical activity with or without their children. Subsequent questions delved into further detail about whether they would continue participating in family-based tag rugby with their children and their enjoyment of the session. Interviews lasted between 5 and 20 min.

#### 2.5.3. Child Interviews

Child interviews were conducted based on family size, and therefore, if one child was present, these were conducted as a single child interview, and if two children were present, these were conducted as a dyadic interview. There were a few instances with three or more children participating at once, and thus, this was conducted as a focus group interview. An opening question asked if children preferred to take part in physical activity with or without their parents. Subsequent questions delved into further detail of whether they would continue participating in family-based tag rugby with their parents, and their enjoyment of the session. Interviews lasted between 5 and 15 min.

### 2.6. Qualitative Content Analysis

Focus groups and interviews were checked against the recordings to ensure accuracy prior to analysis. They were transcribed by the lead researcher and anonymised by removing identifiable characteristics from the data. Transcripts were split into either low, middle, or high socioeconomic status utilising the methods described above, and analysed in the corresponding groups. Qualitative content analysis, with an inductive and semantic approach, was undertaken [33,34]. This analysis was considered most applicable as it allows themes to be identified from the data that are exclusively representative of the perceptions of the participants. Moreover, it is significant for exploration in an area where little is known. The analysis involved a recurring process of immersing oneself in the data and familiarising with the data (preparation phase), rendering the content of the text through the process of coding and classifying themes that denoted similar patterns of communication (organising phase), and reporting the analysis process and results through themes and a storyline (reporting phase) [33]. A coding table was developed and piloted on a subset of transcripts and was continuously refined thereafter in response to emergent data and patterns. The lead researcher engaged in regular peer debriefing to deliberate coding and theme interpretations and to alleviate potential bias. Grouping codes into themes was an iterative process, with close attention to conceptual coherence within groupings and clear differences between them. A detailed examination of language, context, and meaning was applied to guarantee that themes reflected the content and nuances of the transcripts. Direct quotations from participants were systematically extracted to support identified themes. Quotes were selected to illustrate recurring patterns and different perspectives, ensuring that the findings accurately reflect participants’ experiences and perspectives. Theme development was affected by the frequency of occurrence of a topic within the data, which was important in relation to the research question, and included a thorough examination of language and meaning [33,34].

### 2.7. Statistical Analysis

An *a priori* calculation was conducted using G*power 3.1.9.7 and with an alpha probability of 0.05, and based on the previously reported effects of exercise on cognitive function in children (Williams et al., 2020 [17]; Hatch et al., 2021 [35]), an estimated effect size of 0.3 (two-tailed significance), a total sample size of 24 children and 24 parents was required. All data were analysed using SPSS (version 29, SPSS Inc., Chicago, IL, USA). Data were assessed for normality using the Shapiro–Wilk test and visual inspection of histograms and Q–Q plots, which revealed that all data were normally distributed, apart from insulin, which was log-transformed prior to analysis. A two-way repeated measures ANOVA was utilised in children and parents separately to assess the trial*time interactions for postprandial glucose, insulin, and triglycerides, and inverse efficiency scores on the Stroop test, Sternberg paradigm, and Flanker task. Where multiple comparisons were performed, a Bonferroni correction was applied to reduce the risk of type I errors. A paired samples t-test was utilised to assess differences between exercise and rest in children and parents separately for glucose, insulin, and triglyceride iAUC and blood glucose, plasma insulin, and plasma triglyceride peak concentration, and heart rate. An independent samples t-test was utilised for the GPS and heart rate data to assess differences between children and parents during the exercise session. A two-way mixed model ANOVA (age*trial) was utilised to assess the interaction between age (parent or child) and trial (exercise or rest) on glucose, insulin, and triglyceride iAUC and peak values. A three-way mixed model ANOVA (age*trial*time) was utilised to assess the interaction between age (parent or child), trial (exercise or rest), and time on postprandial blood glucose, plasma insulin, and plasma triglycerides, and inverse efficiency scores for the Stroop test, Sternberg paradigm and Flanker task. Partial eta squared was used as the measure of effect size for all analyses (small effect ≥ 0.01; medium effect ≥ 0.06; large effect ≥ 0.14). For all analyses, the alpha level for statistical significance was *p* < 0.05. Data are presented as mean ± SD unless otherwise stated.

## 3. Results

### 3.1. Exercise Characteristics

Average heart rate was higher during the exercise period compared to the rest period in children (exercise: 143 ± 8 beats·min^−1^ vs. rest: 90 ± 4beats·min^−1^; t_22_ = 6.11, *p* < 0.001) and in parents (exercise: 129 ± 8 beats·min^−1^ vs. rest: 77 ± 4 beats·min^−1^; t_10_ = 6.03, *p* < 0.001). Maximum heart rate was higher during the exercise period compared to the rest period in children (exercise: 188 ± 4 beats·min^−1^ vs. rest: 136 ± 7 beats·min^−1^; t_34_ = 6.38, *p* < 0.001) and in parents (exercise: 162 ± 3 beats·min^−1^ vs. rest: 98 ± 5 beats·min^−1^; t_21_ = 10.61, *p* < 0.001). There was no difference in the average heart rate as a percentage of age-predicted maximum heart rate between children and parents during the exercise period (children: 69 ± 4% maximum heart rate (HR_max_), parents: 73 ± 3% HR_max_, *p* = 0.937). There was no difference in the maximum heart rate as a percentage of heart rate max between children and parents during the exercise period (children: 90 ± 2% HR_max_, parents: 92 ± 1% HR_max_, *p* = 0.492).

During the bout of exercise, the average total distance covered by children was 2368 ± 203 m and 2191 ± 318 m in parents. There was no significant difference between the distance covered by children and parents during the 45 min bout of tag rugby (*p* = 0.630).

### 3.2. Cardiometabolic Health Responses

Blood glucose, plasma insulin, and triglyceride concentrations across the exercise and resting trials are presented in Supplementary Table S1a,b.

#### 3.2.1. Blood Glucose Concentrations

*Postprandial Blood Glucose, Glucose iAUC, and Glucose Peak in Children*

**Figure 1:**
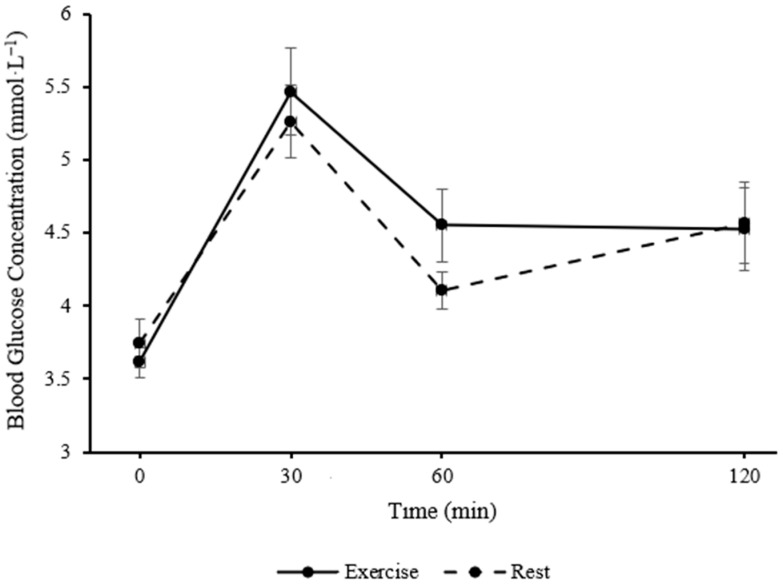
Postprandial blood glucose concentrations for exercise and rested control trials in children. Data are mean ± SEM.

In children, blood glucose concentration was similar between the exercise and rest trials following the consumption of the standardised lunch (main effect of trial; *p* = 0.507) but changed over time (main effect of time; *F*_(3, 39)_ = 16.66, *p* < 0.001, η_p_^2^ = 0.562, large effect, see Figure 1). The pattern of change in blood glucose concentration was similar between the exercise and resting control trial (trial*time interaction; *p* = 0.469).

In children, postprandial glucose iAUC following the standardised lunch did not differ between the exercise and resting control trial (*p* = 0.211), nor were there any differences in peak blood glucose concentrations between trials (*p* = 0.850).

*Postprandial Blood Glucose, Glucose iAUC, and Glucose Peak in Parents*

**Figure 2:**
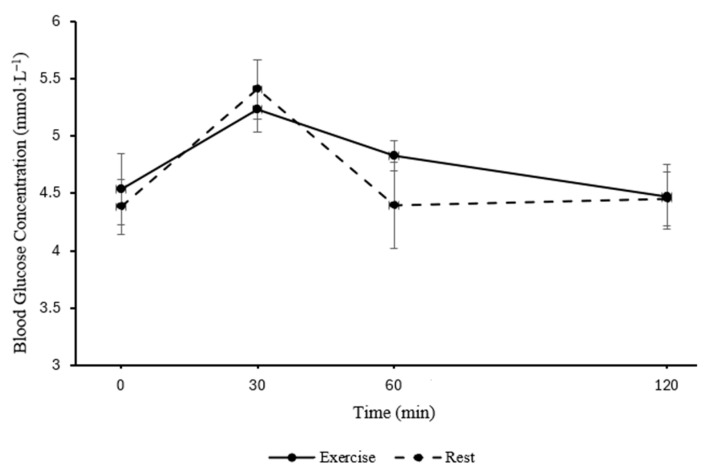
Postprandial blood glucose concentrations for the exercise and rested control trials in parents. Data presented as mean ± SEM.

In parents, blood glucose concentration was similar between the exercise and rest trials following the consumption of the standardised lunch (main effect of trial; *p* = 0.654) but changed over time (main effect of time; *F*_(3, 30)_ = 5.92, *p* = 0.003, η_p_^2^ = 0.372, large effect, see Figure 2). The pattern of change was similar between the exercise and resting control trial (trial*time interaction; *p* = 0.616).

In parents, postprandial glucose iAUC following the standardised lunch did not differ between the exercise and resting control trial (*p* = 0.076), nor were there any differences in peak blood glucose concentrations between trials (*p* = 0.633).

*Family Analysis*

There was no difference in postprandial blood glucose concentration between children and parents across the exercise and resting trial (main effect of age; *p* = 0.126). Furthermore, the postprandial blood glucose response to exercise and rest was similar between children and parents (age*trial interaction; *p* = 0.959), and the pattern of change over time was similar in children and parents (time*age interaction; *p* = 0.053). The effects of exercise and rest did not differ between children and parents over time (trial*time*age interaction; *p* = 0.745).

There was no difference in glucose iAUC between children and parents across the exercise and resting trial (main effect of age; *p* = 0.144). Furthermore, the blood glucose iAUC response to exercise and rest in children and parents was similar (age*trial interaction; *p* = 0.800).

There was no difference in peak blood glucose concentrations between children and parents (main effect of age; *p* = 0.724). Furthermore, the peak blood glucose response to exercise and rest in children and parents was similar (age*trial interaction; *p* = 0.822).

#### 3.2.2. Plasma Insulin Concentrations

*Postprandial Plasma Insulin, Insulin iAUC, and Insulin Peak in Children*

In children, plasma insulin concentration was similar between the exercise and resting trial following the standardised lunch (main effect of trial; *p* = 0.067) but changed over time (main effect of time; *F*_(3, 45)_ = 32.87, *p* < 0.001, η_p_^2^ = 0.687). The pattern of change was similar between the exercise and resting trials (trial*time interaction; *p* = 0.134).

In children, postprandial insulin iAUC following the standardised lunch did not differ between the exercise and resting trial (*p* = 0.712), nor were there any differences in peak plasma insulin concentrations between the exercise and resting trials (*p* = 0.184).

*Postprandial Plasma Insulin, Insulin iAUC, and Insulin Peak in Parents*

**Figure 3:**
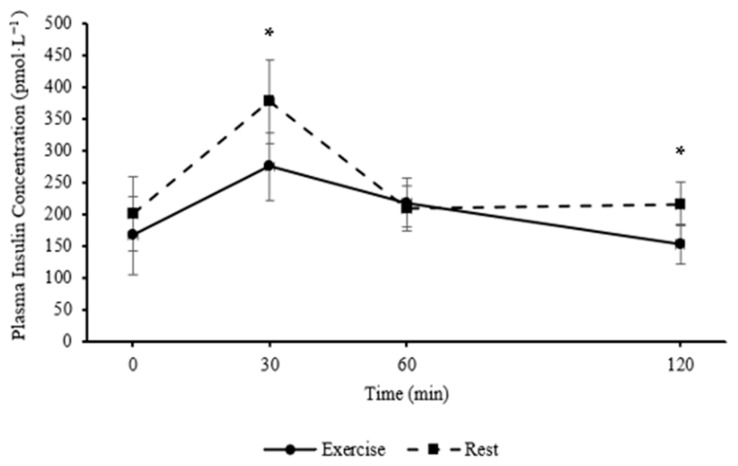
Postprandial insulin concentrations for exercise and rested control trials in parents. Data presented as mean ± SEM. * Significant difference between exercise and rest.

In parents, plasma insulin concentration was lower during the exercise trial compared to the resting trial following the consumption of the standardised lunch (main effect of trial; *F*_(1, 12)_ = 5.32, *p* = 0.040, MD = −0.11 pmol·L^−1^, 95% CI [−0.22, −0.01 pmol·L^−1^]), and changed over time (main effect of time; *F*_(3, 36)_ = 8.67, *p* < 0.001, η_p_^2^ = 0.419, large effect). In parents, the pattern of change differed between the exercise and resting trials (trial*time interaction; *F*_(3, 36)_ = 2.99, *p* = 0.044, η_p_^2^ = 0.200, large effect), whereby plasma insulin concentration was lower 30 min (*p* = 0.004) and 120 min post lunch (*p* = 0.011, Figure 3) in the exercise trial when compared with the rested control trial. While the reduction in insulin following exercise was statistically significant and of large effect size, the acute nature of the session means the chronic physiological implications remain unclear.

In parents, postprandial insulin iAUC following the standardised lunch did not differ between the exercise and resting trial (*p* = 0.459). Peak plasma insulin concentrations following the standardised lunch were lower in the exercise trial compared to the resting trial (t _(12)_ = −3.60, *p* = 0.004, 95% CI [−0.18, −0.04 pmol·L^−1^]).

*Family Analysis*

There was a difference in postprandial plasma insulin concentration between children and parents across the exercise and rested control trial (children: 1.9 pmol·L^−1^, parents: 2.2 pmol·L^−1^; main effect of age; *p* = 0.017), whereby plasma insulin concentration was greater in parents than in children. The postprandial plasma insulin response to exercise and rest was similar between children and parents (age*trial interaction; *p* = 0.941), however the pattern of change over time was different in children and parents (time*age interaction; *F*_(3, 81)_ = 5.01; *p* = 0.003; η_p_^2^ = 0.157, large effect), whereby plasma insulin concentrations were lower in children than in parents at baseline (*p* = 0.003) and 120 min (*p* = 0.035) post lunch across the exercise and rest trials. The effects of exercise and rest were different between children and parents over time (trial*time*age interaction; F _(3, 81)_ = 3.82; *p* = 0.013; η_p_^2^ = 0.124, medium effect), whereby plasma insulin concentrations were lower 30 min (*p* = 0.011) and 120 min (*p* = 0.006) post lunch during the exercise trial compared to the rest trial in parents, whilst the pattern of change in children across time was similar between trials.

There was no difference in insulin iAUC between children and parents across the exercise and resting trial (main effect of age; *p* = 0.671). Furthermore, the response to exercise and rest in children and parents was similar (age*trial interaction; *p* = 0.799).

There was no difference in peak plasma insulin concentrations between children and parents across the exercise and resting trial (main effect of age; *p* = 0.165). Furthermore, the response to exercise and rest in children and parents was similar (age*trial interaction; *p* = 0.321).

#### 3.2.3. Plasma Triglyceride Concentrations

*Postprandial Triglycerides, Triglyceride iAUC, and Triglyceride Peak in Children*

In children, triglyceride concentration was similar between the exercise and resting trial following the standardised lunch (main effect of trial; *p* = 0.251) and did not change over time (main effect of time; *p* = 0.070). Furthermore, the pattern of change was similar between the exercise and resting trials (trial*time interaction; *p* = 0.255).

In children, postprandial triglyceride iAUC following the standardised lunch did not differ between the exercise and resting trial (*p* = 0.373), nor were there any differences in peak triglyceride concentrations between the exercise and resting trials (*p* = 0.393).

*Postprandial Triglycerides, Triglyceride iAUC, and Triglyceride Peak in Parents*

In parents, triglyceride concentration was similar between the exercise and resting trials following the standardised lunch (main effect of trial; *p* = 0.282) but changed over time (main effect of time; *F*_(3, 33)_ = 4.16, *p* = 0.013, η_p_^2^ = 0.274, large effect). Furthermore, the pattern of change was similar between the exercise and resting trials (trial*time interaction; *p* = 0.093).

In parents, postprandial triglyceride iAUC following the standardised lunch did not differ between the exercise and resting trial (*p* = 0.164), nor were there any differences in peak triglyceride concentrations between the exercise and resting trials (*p* = 0.181).

*Family Analysis*

There was no difference in postprandial plasma triglyceride concentration between children and parents across the exercise and resting trial (main effect of age; *p* = 0.129). The postprandial plasma triglyceride response to exercise and rest was similar between children and parents (age*trial interaction; *p* = 0.752), and the pattern of change over time was similar in children and parents (time*age interaction; *p* = 0.707). The effects of exercise and rest did not differ between children and parents over time (trial*time*age interaction; *p* = 0.277).

There was no difference in plasma triglyceride iAUC between children and parents across the exercise and resting trial (main effect of age; *p* = 0.952). Furthermore, the response to exercise and rest in children and parents was similar (age*trial interaction; *p* = 0.106).

There was no difference in triglyceride peak concentrations between children and parents across the exercise and resting trial (main effect of age; *p* = 0.132). Furthermore, the response to exercise and rest in children and parents was similar (age*trial interaction; *p* = 0.713).

### 3.3. Cognitive Function Outcomes

Cognitive function inverse efficiency scores across the exercise and resting trials are presented in Supplementary Table S2.

#### 3.3.1. Stroop Test

*Children*

Overall inverse efficiency scores for the Stroop congruent level were similar between the exercise and rested control trial in children (main effect of trial; *p* = 0.971) and were similar across time (main effect of time; *p* = 0.326). The pattern of change between the exercise and resting trial was also similar (trial*time interaction; *p* = 0.717).

For the Stroop incongruent level, inverse efficiency scores were similar between the exercise and resting trials in children (main effect of trial; *p* = 0.633) and were similar over the course of the day (main effect of time; *p* = 0.319). Furthermore, the pattern of change between the exercise and resting trial was similar (trial*time interaction; *p* = 0.209).

Overall, no significant differences were observed in children’s performance on the Stroop task between exercise and rest.

*Parents*

**Figure 4:**
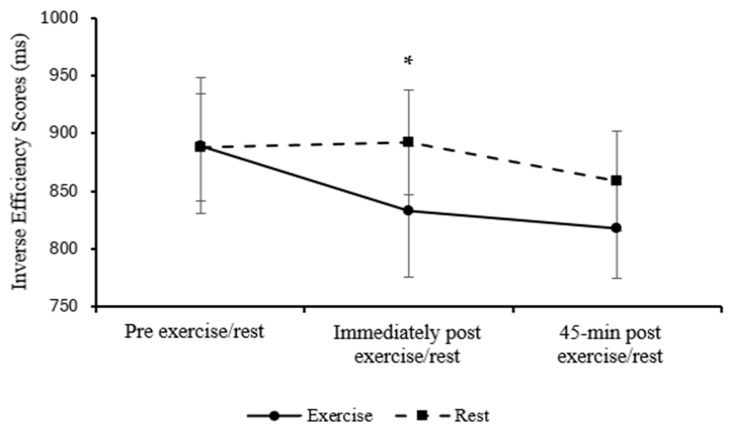
Stroop congruent level inverse efficiency scores (ms) for the exercise and resting control trials in parents. Data are mean ± SEM. * Significant difference between exercise and rest.

Overall inverse efficiency scores for the Stroop congruent level were similar between the exercise and rested control trial in parents (main effect of trial; *p* = 0.129) and improved over the course of the day (main effect of time; *F*_(2, 34)_ = 3.58, *p* = 0.039, η_p_^2^ = 0.174, large effect). Furthermore, the pattern of change differed across trials (trial*time interaction; *F*_(2, 34)_ = 5.07, *p* = 0.012, η_p_^2^ = 0.230, large effect, Figure 4), with an improvement in inverse efficiency scores immediately post-exercise (*p* = 0.016).

Inverse efficiency scores for the Stroop incongruent level were similar between the exercise and rested control trial in parents (main effect of trial; *p* = 0.555) and improved over the course of the day (main effect of time; *F*_(2, 34)_= 6.83, *p* = 0.003, η_p_^2^ = 0.299, large effect). The pattern of change between the exercise and rest trials was similar (trial*time interaction; *p* = 0.134).

Overall, parents’ performance on the congruent level of the Stroop task improved immediately following exercise compared to rest. Their performance on the incongruent level of the Stroop task was not different between exercise and rest, but performance improved over the course of the day.

*Family Analysis*

There was no difference in inverse efficiency scores on the congruent level of the Stroop test between children and parents across the exercise and rest trial (main effect of age; *p* = 0.254). Furthermore, inverse efficiency scores were similar in children and parents between the exercise and resting trial (age*trial interaction; *p* = 0.579), and the pattern of change over the course of the day was similar in children and parents (time*age interaction; *p* = 0.850). The effects of exercise and rest did not differ between children and parents over the course of the day (trial*time*age interaction; *p* = 0.379).

There was no difference in inverse efficiency scores on the incongruent level of the Stroop test between children and parents across the exercise and rest trial (main effect of age; *p* = 0.420). Furthermore, inverse efficiency scores were similar in children and parents between the exercise and rest trial (age*trial interaction; *p* = 0.482), and the pattern of change over the course of the day was similar in children and parents (time*age interaction; *p* = 0.704). The effects of exercise and rest did not differ between children and parents over the course of the day (trial*time*age interaction; *p* = 0.110).

Overall, there were no differences between parents and children, nor any interactions with trial or time, indicating that the pattern of cognitive performance across the Stroop congruent and incongruent tasks was similar across generations and trial conditions.

#### 3.3.2. Sternberg Paradigm

*Children*

Inverse efficiency scores for the Sternberg one-item level were similar between the exercise and rest trials in children (main effect of trial; *p* = 0.662) and were similar over the course of the day (main effect of time; *p* = 0.716). The pattern of change between the exercise and rest trials was similar (trial*time interaction; *p* = 0.071).

**Figure 5:**
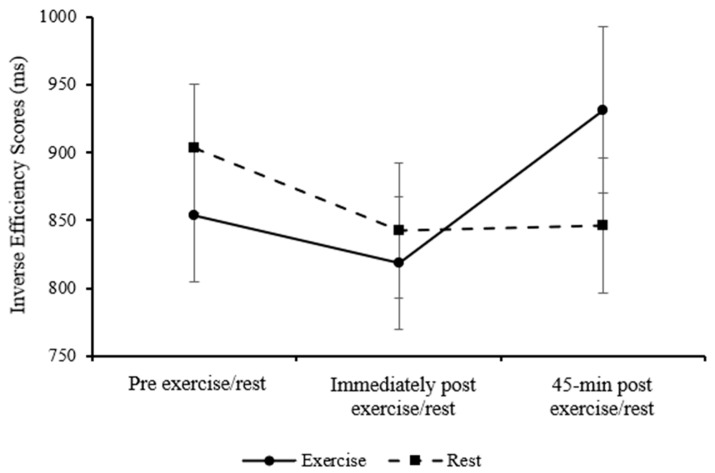
Sternberg three-item inverse efficiency scores (ms) for the exercise and resting control trials in children. Data are mean ± SEM.

For the Sternberg three-item level, inverse efficiency scores were similar between the exercise and rest trials in children (main effect of trial; *p* = 0.872) and were similar over the course of the day (main effect of time; *p* = 0.104). The pattern of change between the exercise and rest trial differed (trial*time interaction; *F*_(2, 42)_ = 4.60, *p* = 0.016, η_p_^2^ = 0.180, large effect, Figure 5); however, inverse efficiency scores immediately post-exercise and 45 min post-exercise were not statistically significant (immediately post-exercise, *p* = 0.489; 45 min post-exercise, *p* = 0.093). Precisely, children’s working memory performance improved immediately following exercise compared to rest, yet worsened 45 min post-exercise compared to rest, suggesting that the beneficial effects of exercise on children’s cognition may be transient and diminish over time.

Inverse efficiency scores for the Sternberg five-item level were similar between the exercise and rest trials in children (main effect of trial; *p* = 0.581) and improved immediately post-exercise or rest, and worsened 45 min post-exercise and rest (main effect of time; *p* = 0.030). Furthermore, the pattern of change between the exercise and rest trials was similar (trial*time interaction; *p* = 0.255).

Overall, for the one-item and five-item levels, no significant differences were observed in children’s performance. For the three-item level, children’s performance improved immediately following exercise and worsened 45 min following exercise when compared with rest.

*Parents*

**Figure 6:**
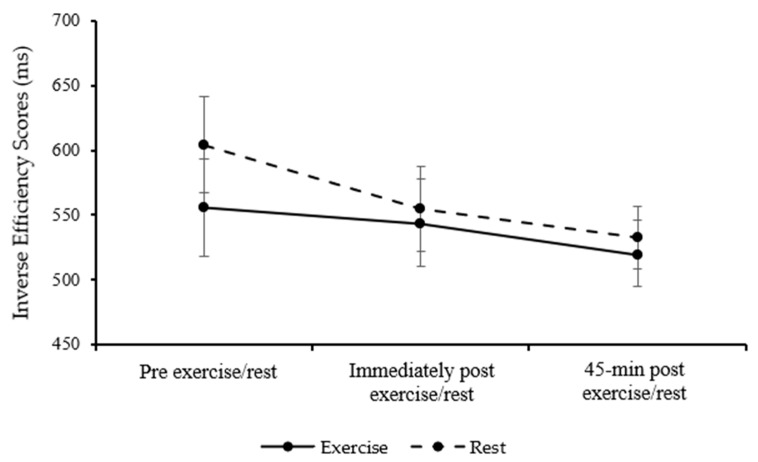
Sternberg one-item inverse efficiency scores (ms) for the exercise and resting control trials in parents. Data are mean ± SEM.

Inverse efficiency scores for the Sternberg one-item level were similar between the exercise and rest trial in parents (main effect of trial; *p* = 0.122) and improved over the course of the day (main effect of time; *F*_(2,32)_= 6.83, *p* = 0.014, η_p_^2^ = 0.233, large effect, Figure 6). The pattern of change between the exercise and rest trials was similar (trial*time interaction; *p* = 0.423).

**Figure 7:**
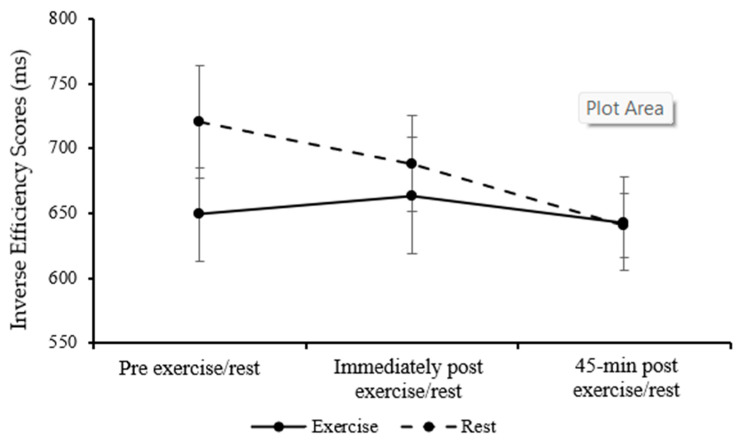
Sternberg three-item inverse efficiency scores (ms) for the exercise and resting control trials in parents. Data are mean ± SEM.

For the Sternberg three-item level, inverse efficiency scores were similar between the exercise and rest trial in parents (main effect of trial; *p* = 0.116) and improved over the course of the day (main effect of time; *F*_(2,32)_= 6.83; *p* = 0.016; η_p_^2^ = 0.226, large effect, Figure 7). The pattern of change between the exercise and rest trials was similar (trial*time interaction; *p* = 0.311).

Inverse efficiency scores for the Sternberg five-item level were similar between the exercise and rest trial in parents (main effect of trial; *p* = 0.373) and were similar over the course of the day (main effect of time; *p* = 0.167). The pattern of change between the exercise and rest trials was similar (trial*time interaction; *p* = 0.951).

Overall, parents’ performance on the Sternberg paradigm was not different between exercise and rest but did improve over the course of the day for the one-item and three-item levels.

*Family Analysis*

There were differences in inverse efficiency scores on the Sternberg one-item level between children and parents across the exercise and rest trial (children: 661.4 ms, parents: 543.3 ms; main effect of age; *p* = 0.013), whereby parents’ performance was significantly better than children’s. However, inverse efficiency scores were similar in children and parents between the exercise and rest trial (age*trial interaction; *p* = 0.366), as was the pattern of change over the course of the day (time*age interaction; *p* = 0.593). The effects of exercise and rest did not differ between children and parents across time (trial*time*age interaction; *p* = 0.066).

There was a difference in inverse efficiency scores on the three-item Sternberg level between children and parents (children: 872.3 ms, parents: 656.9 ms; main effect of age; *p* = 0.002), whereby parents’ performance was significantly better than children’s. Inverse efficiency scores were similar in children and parents between the exercise and rest trial (age*trial interaction; *p* = 0.519). Yet, the pattern of change across time was different in children and parents (time*age interaction; *F*_(2, 74)_ = 4.10, *p* = 0.022, η_p_^2^ = 0.098, medium effect), whereby children’s inverse efficiency scores were lower immediately following exercise and rest. However, children’s inverse efficiency scores increased 45 min post-exercise or rest, whilst parents’ scores remained low. The effects of exercise and rest did not differ between children and parents over time (trial*time*age interaction; *p* = 0.397).

There was a difference in inverse efficiency scores on the five-item Sternberg level between children and parents (children: 1081.5 ms, parents: 790.5 ms; main effect of age; *p* < 0.001), whereby parents’ performance was significantly better than children’s. Furthermore, inverse efficiency scores were similar in children and parents between the exercise and rest trial (age*trial interaction; *p* = 0.327), and the pattern of change over time was different in children and parents (time*age interaction; *F*_(2, 72)_ = 4.60 *p* = 0.013, η_p_^2^ = 0.113, medium effect), whereby children’s inverse efficiency scores were lower immediately following exercise and rest but increased 45 min post-exercise or rest. Parents’ inverse efficiency scores were higher immediately following exercise/rest and decreased 45 min post-exercise/rest. The effects of exercise and rest did not differ between children and parents over time (trial*time*age interaction; *p* = 0.310).

Overall, parents’ performance was significantly better across all levels of the Sternberg paradigm, and children and parents respond differently over time for the three-item and five-item levels. There were no interactions with trial for all levels, indicating that exercise did not differentially affect working memory across generations.

#### 3.3.3. Flanker Task

*Children*

Overall inverse efficiency scores for the Flanker congruent level were similar between the exercise and rest trials in children (main effect of trial; *p* = 0.207) and were similar across time (main effect of time; *p* = 0.500). The pattern of change between the exercise and rest trial was similar (trial*time interaction; *p* = 0.610).

Inverse efficiency scores for the Flanker incongruent level were similar between the exercise and rest trials in children (main effect of trial; *p* = 0.420) and were similar across time (main effect of time; *p* = 0.279). The pattern of change between the exercise and rest trial was similar (trial*time interaction; *p* = 0.358).

Overall, for the Flanker task, no significant differences were observed in children’s performance.

*Parents*

Overall inverse efficiency scores for the Flanker congruent level were similar between the exercise and rest trials in parents (main effect of trial; *p* = 0.665) and were similar across time (main effect of time; *p* = 0.204). Furthermore, the pattern of change between the exercise and resting trial was similar (trial *time interaction; *p* = 0.242).

Inverse efficiency scores for the Flanker incongruent level were similar between the exercise and rest trials in parents (main effect of trial; *p* = 0.384) and were similar across time (main effect of time; *p* = 0.657). Furthermore, the pattern of change between the exercise and rest trial was similar (trial*time interaction; *p* = 0.106).

Overall, for the Flanker task, no significant differences were observed in parents’ performance.

*Family Analysis*

There was a difference in inverse efficiency scores on the congruent level of the Flanker task between children and parents (main effect of age; *p* = 0.020), whereby children’s inverse efficiency scores were higher than their parents. Yet, inverse efficiency scores were similar in children and parents between the exercise and rest trial (age*trial interaction; *p* = 0.213), and the pattern of change across time was similar in children and parents (time*age interaction; *p* = 0.931). The effects of exercise and rest did not differ between children and parents across time (trial*time*age interaction; *p* = 0.274).

There was no difference in inverse efficiency scores on the incongruent level of the Flanker task between children and parents (main effect of age; *p* = 0.066). Furthermore, inverse efficiency scores were similar in children and parents between the exercise and rest trial (age*trial interaction; *p* = 0.516), and the pattern of change over the course of the day was similar in children and parents (time*age interaction; *p* = 0.346). The effects of exercise and rest did not differ between children and parents over the course of the day (trial*time*age interaction; *p* = 0.281).

Overall, there were no differences between parents and children, nor any interactions with trial or time, indicating that the pattern of cognitive performance across the Flanker task was similar across generations and trial conditions.

### 3.4. Focus Groups and Interviews

Families highlighted several factors that shaped their perceptions of their enjoyment of the tag rugby session and the feasibility of implementing tag rugby at home. Specifically, two themes were developed for enjoyment: inclusivity and whole family enjoyment, and engaging elements that captivate children and parents. Three themes were developed for feasibility: modality, intensity, and session duration, integrating family tag rugby into family life, and traditional low-impact sports as alternatives.

Illustrative quotes are presented in Supplementary Table S3, with a focus within the text on themes which were most prevalent in the focus groups/interviews and most significant in their impact.

#### 3.4.1. Families Perceived Enjoyment of Tag Rugby

Overall, families felt the tag rugby session provided an inclusive environment for children and their parents to exercise, and the enjoyment of the session outweighed the fact that they were exercising. Furthermore, tag rugby was highlighted as an exercise modality that parents and their children can, and want to, participate in together.

*Inclusive and Enjoyable for the Whole Family*

All families, regardless of socioeconomic status, felt that the enjoyment of the tag rugby session surpassed the fact that they were exercising. One mother from the low socioeconomic status group reflected that tag rugby brings an enjoyable aspect to physical activity.

*“But they [daughters] tend to moan a lot, you know, like oh you know, my legs are hurting. When are we there yet? So I think the tag rugby thing brings the fun aspect to it where they don’t actually realise that they’re exercising. Yeah, because they’re enjoying what they’re doing, you know?”* (Mother, low socioeconomic status)

When discussing the tag rugby session with parents alone, regardless of their socioeconomic status, most suggested they preferred to participate in physical activity without their children, in general, due to factors such as differing abilities and interests, and wanting time to themselves. However, they felt that tag rugby created an inclusive environment for the whole family, which provided a novel, interactive, and viable opportunity to exercise as a family.

*“I think it was nice for me to do that with the children. Yeah, because I guess there are sports that the children do where they’re doing it themselves and you’re watching them, aren’t you? So it’s quite a nice activity to get involved in where yeah, you know you have enjoyed and have fun together. Yeah. So, yeah, I thought it was, it was nice”* (Mother, low socioeconomic status)

*“Yeah, I mean the weather was great and there were different tasks and it was interactive with ages and the families as well, so it was good”* (Mother, middle socioeconomic status)

Similarly, children, regardless of their socioeconomic status, suggested that they typically preferred to participate in physical activity without their parents. Following this, they implied that they would like to participate in tag rugby again with their parents, as it was fun and novel.

*“I think it depends what it is [mode of physical activity], because if it was like that fun tag rugby then that’s fine. If it was at like a different level, then I’d rather do it myself, yeah”* (Daughter, high socioeconomic status)

Furthermore, all children reflected on the opportunities available to them for family physical activity and felt that tag rugby provided an opportunity for them to exercise as a unit, something they would not normally do.

*“I think it was fun because we don’t normally do stuff like that with each other, so it’s like very different”* (daughter, high socioeconomic status)

*Engaging Elements that Captivate Children and their Parents*

Parents from the low socioeconomic group felt their children were faster and more capable during the tag rugby small-sided games; however, this was deemed as encouraging for parents and a facilitator for future participation.

*“It was challenging because they’re kids, you know, they’re faster than that. So yeah, they’ve got more energy, though they’re pushing us”* (Mother, low socioeconomic status)

Conversely, parents in the middle-high socioeconomic groups felt that even though the session was fast-moving, they were able to keep up with their children during the session. Additionally, they felt the fast-paced nature of the session kept everyone engaged.

*“I thought it was good because it was quite fast moving, and so it kept everyone interested. You were just, before you could get bored anything like that in terms of switching it kept everybody on their toes and kept everybody engaged so that was quite good”* (Mother, middle socioeconomic status)

Regardless of socioeconomic status, most parents preferred the organised drill component of the tag rugby session. They suggested that this provided a structured environment for them to exercise, further suggesting that the small-sided games were “not well organised” (Mother, low socioeconomic status). Parents in the middle–high socioeconomic groups suggested that including more rules during the small-sided games would maintain structure so that children would not misbehave and interrupt the session.

*“It [tag rugby game] almost got too, too free, but then some of us wanted to bring it back a little bit whereas the kids just wanted to carry on being silly. Not that it was a bad thing to be silly, we were just thinking more of the game”* (mother, high socioeconomic status)

Conversely, children from all socioeconomic groups preferred the small-sided games and described the drill component as repetitive, which hindered enjoyment.

*“I liked the drills except when it got repetitive, so for quite a long time it didn’t feel as fun”* (Son, high socioeconomic status)

They further explained that the small-sided games were relaxed and non-competitive, allowing them to focus on having fun.

*“It [tag rugby game] was very very fun. It didn’t feel that competitive. I felt like I was more having fun than competitive”* (Son, high socioeconomic status)

Thus, there seems to be a generational difference in preferences; parents favour organised components with more structure, while children prefer a more relaxed approach. Future interventions should offer a variety of activities that incorporate structured drills with casual game elements, catering to children and parents.

#### 3.4.2. Feasibility of Implementing Family-Based Tag Rugby at Home

Overall, families from all socioeconomic groups felt that 45 min of tag rugby, 1–3 times per week, would feasibly work for them, whilst perceptions of the intensity of the session varied across generations, as well as across socioeconomic groups.

*Modality, Intensity, and Duration of the Session*

All families, regardless of socioeconomic status, acknowledged that the 45 min tag rugby session was not overly time-consuming. They further expressed that the duration of the session was right for them, and parents from the low socioeconomic status group felt that the inclusion of breaks was ideal.

*“No, it was ok because, because there was, there was a, there was half time, so there was half time. So, it’s ok when doing it, she gave us a minute. Then we continue”* (Mother, low socioeconomic status)

Families from the low socioeconomic group described the flexible nature of tag rugby by discussing how it can be adapted to different settings, including indoors or outdoors, and can be inclusive for multiple families, too. They further suggested that this would allow friendships between families to form, and highlighted that it is a form of physical activity that can be utilised to break the day up.

*“Similar to how you might go out and walk the dog, you could be with your kids on the field playing something like that. Couldn’t you, just to break the day up or something? You know, for half an hour or whatever”* (Mother, low socioeconomic status)

*“For me, I think it’s nice to do it with multiple families. There’s more interaction and the kids can have friends, and you know, we can make friends as well”* (Mother, low socioeconomic status)

When discussing the intensity of the session, parents from the low socioeconomic status group found the intensity to be just right, whereas middle–high class parents, and children from all socioeconomic groups felt they could have performed more. Children preferred that the session included “more running around” (son, low socioeconomic status); however, they explained that the session was tiring.

*“I think it could’ve been a bit more high intensity just for myself because, erm, I didn’t feel like I was going as fast as I could’ve but it was tiring”* (Son, high socioeconomic status)

Parents from the middle socioeconomic group suggested that the pitch area for the small-sided games could have been bigger to encourage them to work harder.

*“You could make the pitch a bit bigger so that there was more intensity encouraged and more going on, I think that might help”* (Mother, middle SES)

Finally, all families deemed tag rugby to be an outdoor sport, and thus participating outside, particularly during the warmer, lighter summer months, was most favourable.

*“Yeah, especially in the summer when it’s light and you can go out and enjoy the weather”* (Father, high socioeconomic status)

*Integrating Tag Rugby into Family Life*

All families discussed life commitments as a barrier to participating in physical activity together, and particularly for implementing family tag rugby at home. While most children explained that they participate in physical activity regularly when under the care of their parents, parents identified responsibilities, including work commitments and family duties, as barriers to tag rugby implementation at home.

*“It depends what world we’re living in, if I could be doing something with him once a day I would, but in the reality we are living in with school, other extracurriculars, we could only fit in once a week”* (Mother, high socioeconomic status)

However, families believed they would want to participate in tag rugby frequently and suggested that one to three times a week would be feasible. Specifically, the mornings of weekends were most desirable to participate in family tag rugby, and after school or work were considered by some families, too.

*“Ermm, mornings like after I wake up after breakfast. Erm, because I think morning is when I, I feel more energetic, and it works better”* (Son, middle socioeconomic status)

Therefore, future interventions should recognise these barriers whilst personalising the approach for each family, to ensure the inclusion of both children and their parents.

*Traditional team sports and low-impact sports as alternatives to tag rugby*

Families discussed possible alternatives to family-based tag rugby and reflected on past opportunities they had taken for family physical activity. Two approaches were discussed by all families, which included team-based and low-impact sports.

Families discussed alternatives to family-based tag rugby, and not surprisingly, the most favoured were traditional team sports such as football, basketball, dodgeball, and netball, which were most favoured by children. The children suggested that these were the sports they were most familiar with and interested in.

*“Because I quite like dodgeball and I quite like playing with my parents, so that would be quite fun”* (son, high socioeconomic status)

Conversely, parents spoke of their experiences of participating in physical activity with their children, and contrary to team-based sports, the most familiar were low-impact sports such as walking, cycling, or swimming.

*“So going for walks in the countryside would be my top one because they’re really chilled out. And they get on really well, and they laugh a lot when we do go for walks. Yeah, I know they love going swimming into water parks. I really don’t like it, so I wish I liked swimming more. Whenever you say, what do you want to do? They’ll say family swim, which involves me going on slides and stuff. Erm, yeah, which I’m a bit funny about water, I don’t really like the water. Erm, but yeah, that that would if I if I could get around it that would be a good one for us”* (Mother, high socioeconomic status)

They further explained that family physical activity can be combined with other elements, turning it into a family day out that includes alternative ways of engagement and bonding, such as visiting restaurants or playing card games.

*“And then especially if you can mix it in with going for pub lunch and taking, like, card games. So, that, that would be my favourite thing”* (Mother, high socioeconomic status)

Thus, future interventions could consider games-based alternatives to tag rugby. Furthermore, including aspects beyond physical activity (such as family activities following a family-based physical activity session) could be considered to aid with motivation.

## 4. Discussion

The key findings of the present study are that an acute bout of family tag rugby elicited cardiometabolic health benefits in parents, but not in their children. Another key finding of the present study was that children and their parents displayed cognitive benefits following the acute bout of exercise. When considering responses to tag rugby across generations, children and their parents exhibited similar postprandial glycaemic and lipaemic responses. However, children and their parents exhibited different postprandial insulinaemic responses; parents exhibited a more favourable postprandial insulin response following exercise compared to rest, whereas children showed no such response. There were differences in working memory performance between children and their parents, whereby parents’ performance was significantly better than children’s. During the focus groups and interviews, families deemed tag rugby an ecologically valid and inclusive mode of physical activity, which entails elements that are appealing across generations. Additionally, families from low socioeconomic backgrounds consider tag rugby flexible in nature, which can be adapted to overcome the barriers associated with access to local facilities. The present study provides novel information related to the acute cardiometabolic and cognitive effects of family physical activity in children and their parents, and important considerations for the design of chronic family physical activity interventions.

The present study is the first to examine the acute effects of a bout of family physical activity on cardiometabolic disease risk factors in children and their parents simultaneously. The results indicate that the postprandial insulinaemic and insulin peak responses to an ecologically valid meal following exercise were lower in parents when compared with rest, but not in children. This is an important finding given the crucial role that postprandial insulinaemia plays in the development of insulin resistance and cardiovascular disease risk [36]. A possible explanation for exercise eliciting such effects in parents, but not in children, could be that adults tend to have a greater age-related risk accumulation for insulin resistance involving longer periods of exposure to poor lifestyle factors [37,38,39] and thus greater potential for physical activity to enhance their cardiometabolic health [40]. In contrast, children generally maintain a naturally more favourable cardiometabolic risk profile due to less time exposed to poor lifestyle factors [41]. Prior literature in children has demonstrated that postprandial insulin iAUC was 35% lower following exercise (60 min of basketball) compared to seated rest [16]. However, this study involved only children participating without their parents, who exercised at a greater intensity (96 ± 4% HR_max_) when compared with the present study (90 ± 2% HR_max_). Despite similar engagement with the session, as per the distances covered and percentage of maximum heart rate in children and their parents, parents demonstrated favourable postprandial insulinaemic and insulin peak responses following exercise, whereas children did not. These findings are in line with prior research examining family physical activity interventions, whereby the exercise had no benefit on children’s physical activity levels or traditional cardiometabolic disease risk markers [42]. Future research should consider populations of children who are at risk of an adverse cardiometabolic disease profile, including those with excess weight/obesity, who, in line with parents, may exhibit a greater capacity for improvement following acute exercise. This supports a shift in focus away from generally healthy children toward those at elevated risk, thereby prioritising targeted interventions before transitioning to the development of chronic interventions.

The present study is the first to investigate whether cardiometabolic responses following the standardised lunch exhibit similar trends across generations in the exercise and rest trials. The findings suggest that children and their parents differ in overall postprandial insulin concentrations, that these differences vary depending on the trial condition, and that the pattern of postprandial insulin response over time to exercise and rest differs between children and parents. Mechanisms explaining the differences between children and parents may relate to age-related differences in insulin sensitivity and metabolic maturity. Parents have greater cumulative exposure to poor lifestyle factors and a higher age-related susceptibility to insulin resistance, which may increase their potential to benefit from the effects of physical activity [37,38,39,40]. These mechanisms remain speculative and warrant further investigation. Moreover, qualitative data indicated that parents perceived the session as fast-moving, suggesting that they may have been working harder during the activity. This heightened effort could partly account for why parents showed reductions in postprandial insulin. The findings from the present study further infer that children and their parents respond similarly to the exercise and rest trials when considering postprandial glucose, triglyceride responses, glucose, insulin, and triglyceride iAUC and peak concentrations. Mechanisms to support this suggest that glucose, insulin, and triglyceride metabolism are highly heritable traits [43]. Families often possess similar glucose, insulin, and triglyceride profiles, and the parent–child dyad suggests children and their parents adopt similar lifestyle habits [44]. In turn, these lifestyle habits influence glucose, insulin, and triglyceride metabolism in children and their parents.

Following the acute bout of family tag rugby, children’s working memory improved immediately post-exercise and then worsened 45 min post-exercise when compared to rest, highlighting the transient nature of acute exercise effects on working memory in children, which is consistent in the literature [17,45,46]. The exercise-induced effects on cognition are domain and time-sensitive, with improvements to some domains presenting immediately, and others presenting after a delay [17]. The effects on working memory following exercise could, therefore, be influenced by the delay between the end of exercise and the administration of the cognitive tests. The differences observed in children’s and parents’ working memory performance following exercise may be partly due to the cognitive demands of the tag rugby session. Qualitative findings suggest that parents preferred structured drills, whereas children enjoyed the small-sided games, indicating that parents may favour cognitively engaging physical activity, whereas children may experience greater cognitive fatigue from these approaches. These findings align with theories of exercise-induced cognitive enhancement, where task complexity and cognitive load can modulate executive function benefits [47]. In parents, information processing improved immediately post-exercise and up to 45 min post-exercise when compared with rest. Previous studies have demonstrated that information processing is acutely enhanced by a single bout of exercise in adults [22], and moreover, chronically enhanced following cardiovascular and aerobic-like physical activity programmes [48,49]. The present study is novel in suggesting that an acute bout of family physical activity induces cognitive benefits up to 45 min following exercise in parents. Furthermore, further family-based interventions are yet to consider cognitive outcomes in parents [42]. Thus, warranting further investigation into the chronic effects of family-based physical activity on parents’ cognitive function, given the importance of this for the prevention of cognitive dysfunction, and for enhanced health [50,51]. The present study also identified different patterns across generations for the Stroop test and Flanker task, which may relate to the distinct executive functions each task engages and developmental differences between children and parents. The Stroop test places greater demand on inhibitory control, which develops later in adolescence [52], whereas the Flanker task primarily relies on attentional control, which matures earlier [53].

The present study is the first to investigate families’ perceptions of their enjoyment of a single bout of family-based physical activity, as well as factors they believe would affect the feasibility of implementing family tag rugby at home. The findings respond to the need for evidence of family perceptions following a single taster session of co-participatory exercise, which is necessary to inform future chronic interventions. Overall, families agreed that tag rugby is an inclusive and enjoyable mode of physical activity that children and their parents want to participate in together. All parents felt that the session established an inclusive environment for them to exercise with their children, and provided a novel, interactive, and viable opportunity to exercise as a family. Similarly, all children agreed that tag rugby provides a fun and novel opportunity to exercise with their parents. These findings support previous research, which has recognised that children and parents value enjoyment during a family-based intervention [42] and extend upon them by detailing families’ perceived enjoyment following a single bout of exercise, as opposed to a six-week period of participation [42]. Additionally, the present study provides evidence that not only does family-based tag rugby provide benefits for certain health and cognitive outcomes in children and their parents, but it is also an enjoyable mode of activity with potential for long-term implementation. Moreover, family-based tag rugby provided appealing elements across generations, whereby parents preferred the organised drill components, and children preferred the small-sided games. Together, these findings suggest that family tag rugby is an ecologically valid mode of physical activity for children and their parents, whereby future interventions should incorporate a range of activities that include structured drills and casual game elements to factor in enjoyment across generations. Therefore, for most families, participation in family-based exercise that has elements appealing to children and their parents is likely to promote positive perceptions and motivation towards regular participation.

Although families from middle-to-high socioeconomic backgrounds reported that the session felt low in intensity, heart rate data indicate that both children and parents reached a high percentage of their HRmax (90 ± 2% HR_max_ and 92 ± 1% HR_max_, respectively), suggesting that the session was physiologically demanding, supporting its potential to drive meaningful metabolic and cognitive effects. Importantly, children and their parents, regardless of socioeconomic status, deemed the intensity of the 45 min bout of family-based tag rugby to be acceptable. Furthermore, all families felt that 45 min of tag rugby was not overly time consuming, and participating up to three times per week would be ideal. Thus, the present study offers key insights for the design of future interventions, which should consider implementing 45 min bouts of family-based exercise up to three times per week. Correspondingly to prior work, families discussed life and family commitments as potential barriers to family-based physical activity, and more generally, such barriers have been discussed within research concerning parental physical activity participation alone [54,55]. Thus, the findings in the present study support those of previous studies, and therefore, these barriers to physical activity participation should be considered moving forward. Future work might also consider investigating approaches to reduce such barriers to physical activity participation.

A recognised barrier to family-based physical activity participation for low socioeconomic families is access to and provision of local authority and privately owned leisure centres [28]. In the present study, low socioeconomic status families described the flexible nature of tag rugby, discussing that tag rugby can be adapted to different indoor and outdoor settings. This suggests that tag rugby is adaptable in nature and could be utilised to overcome barriers associated with access to and provision of local facilities. This is promising as prior work has suggested utilising a mode of physical activity that can be performed indoors or outdoors, so that families from a range of socioeconomic backgrounds can participate.

Crucially, parents from the low socioeconomic status group felt that their children were faster and more capable during the small-sided tag rugby games, but deemed this to be encouraging and a facilitator for future participation. Like previous findings, low socioeconomic status parents discussed their children as role models for physical activity participation, and because they perceived their children as physically fitter, their desire to continue participating in physical activity derived from feeling like they want to be able to keep up with their children [28]. These prior perceptions were discussed as facilitators prior to family-based physical activity participation. Thus, the present study builds on this knowledge to portray that child role modelling can actively encourage parents during a family-based physical activity session. Again, this is promising for motivation towards regular physical activity participation.

The present study has many strengths; it is the first to consider cardiometabolic disease risk factors beyond blood pressure and cognition in children and their parents in response to exercising together. Additionally, it is the first to consider whether children and their parents exhibit similar trends in cardiometabolic responses to exercise and rest. Furthermore, it is the first to reflect on families’ perceptions of their enjoyment of the acute bout of exercise prior to a long-term family-based intervention, whilst also considering families’ perceptions of the feasibility of implementing tag rugby at home. Additionally, the present study considered how qualitative perceptions of the acute family-based physical activity session differ across socioeconomic status. Finally, a great strength of the present study is its robust design and control of variables (such as dietary intake), which have the potential to impact cardiometabolic responses and the exercise–cognition relationship. Although the present study has many strengths, it also has limitations; the effects of participation in a single bout of family-based physical activity on cardiometabolic health and cognitive function were only examined acutely, and thus long-term effects (including up to 24 h post-exercise) remain unknown. Similarly with studies of this nature, families that participated in the present study are not representative of all families; there is an inclination for more physically active families to participate in exercise research. Additional limitations include the introduction of possible bias by the small and potentially more active sample, potential influences of participants’ motivation, and prior activity levels. We also relied on participants to self-report their dietary intake so that diet could be matched prior to the main trials; this method may introduce recall inaccuracies. We required 24 parents to participate in the present study; however, only 20 participated. Due to this shortfall, the statistical power of the analyses may be reduced and thus increase the likelihood of failing to detect small to moderate effects. We did not include an objective measure of session intensity and therefore relied on heart rate and GPS data to quantify session intensity. As most families reported having no prior experience with tag rugby, the qualitative findings may be influenced by novelty effects. Given that the study examined responses to a single acute session, the generalisability of the findings is limited. Finally, due to issues with organising data collection sessions, some families completed the exercise component of the study as a single family unit, whilst some completed this with multiple families, which may have influenced participants’ perceptions of the session.

## 5. Conclusions

Overall, the present study suggests that a single bout of family tag rugby can elicit short-term cardiometabolic benefits in parents and cognitive benefits in children and parents, while also being an enjoyable form of activity with potential for chronic implementation and participation. These findings provide initial insight into the acute responses of children and parents to family-based physical activity. Parents showed improvements in insulin responses, whereas children did not, though glycaemic and lipaemic responses were similar between generations. Cognitive benefits were immediate for children and persisted up to 45 min post-exercise for parents. Qualitative findings indicate that families from a range of socioeconomic backgrounds perceive tag rugby as ecologically valid, adaptable, and appealing across generations. Crucially, these results reflect acute responses to a single session of family-based physical activity, and further research is needed to determine whether chronic or repeated participation would produce sustained cardiometabolic and cognitive adaptations.

## Figures and Tables

**Table 1:** Descriptive overview of the number of families, children, and parents, and respective socioeconomic status groups.

Socioeconomic Status Group	Number of Families	Number of Children	Number of Parents
Low Socioeconomic Status (Deciles 1–3)	6	8	6
Middle Socioeconomic Status (Deciles 4–7)	5	11	8
High Socioeconomic Status (Deciles 8–10)	5	8	6

Socioeconomic status determined via the UK Indices of Multiple Deprivation 2019 (Ministry of Housing, Communities and Local Government, 2019 [30]).

**Table 2:** Descriptive participant characteristics of children and parents. Data are presented as mean ± SD.

Characteristic	Children			Parents		
	Overall *N* = 27	Girls *n* = 11	Boys *n* = 16	Overall *N* = 20	Mothers *n* = 14	Fathers *n* = 6
Age (y)	11.73 ± 1.93	11.91 ± 2.13	11.60 ± 1.85	45.50 ± 9.02	44.08 ± 8.84	48.83 ± 9.33
Height (m)	1.50 ± 0.13	1.51 ± 0.14	1.50 ± 0.13	1.69 ± 0.06	1.67 ± 0.04	1.75 ± 0.05
Body mass (kg)	44.67 ± 14.36	42.93 ± 16.44	45.87 ± 13.16	86.51 ± 20.39	82.88 ± 22.30	94.98 ± 12.83
BMI (kg/m^2^)	19.58 ± 5.05	18.40 ± 4.70	20.40 ± 5.26	30.22 ± 6.89	29.78 ± 7.79	31.24 ± 4.58
BMI Percentile	45.26 ± 35.57	29.91 ± 29.61	55.81 ± 36.30	N/A	N/A	N/A
Maturity Offset (y)	−1.10 ± 1.95	−0.03 ± 2.03	−1.84 ± 1.55	N/A	N/A	N/A

N/A refers to that these measures are not applicable to parents.

## Data Availability

The data presented in this study are available on request from the corresponding author due to the inclusion of participants’ or their guardians’ personal privacy information.

## Supplementary Materials

The following supporting information can be downloaded at: https://www.mdpi.com/article/10.3390/healthcare13243186/s1. Table S1a: Blood glucose, plasma insulin, and plasma triglyceride concentrations in children and parents across the exercise trial. Data are mean ± SD. Table S1b: Blood glucose, plasma insulin, and plasma triglyceride concentrations in children and parents across the rest trial. Table S2: Inverse efficiency scores (ms) for the Stroop test, Sternberg paradigm, and Flanker task in children and parents across the exercise and resting trials. Data are mean ± SD. Table S3: Themes and sub-themes representing factors of families’ enjoyment and the feasibility of implementing tag rugby at home, with exemplar quotes.
