# Supplementary material for: Family-Based Tag Rugby: Acute Effects on Risk Factors for Cardiometabolic Disease and Cognition and Factors Affecting Family Enjoyment and Feasibility
Source: Healthcare (Basel). 2025 Dec 5;13(24):3186. doi: 10.3390/healthcare13243186 (PMC12733199; doi:10.3390/healthcare13243186)
Supplement: Supplementary file 1 [file healthcare-13-03186-s001.zip › healthcare-3990063-supplementary.pdf]

Supplementary Materials

Table S1a. Blood glucose, plasma insulin and plasma triglyceride concentrations in children and parents across the exercise trial. Data are mean ± SD.

		Exercise					
**Variable**	**Group**	**Baseline**	**30-min postprandial**	**60-min postprandial**	**120-min postprandial**	**Peak Concentration**	**iAUC**
**Blood Glucose (mmol·L^{-1})**	**Children**	3.61 ± 0.39	5.47 ± 1.10	4.55 ± 0.93	4.52 ± 1.05	5.71 ± 0.90	135.13 ± 72.33
	**Parents**	4.53 ± 1.03	5.23 ± 0.66	4.83 ± 0.44	4.47 ± 0.95	5.68 ± 0.77	105.57 ± 60.05
**Plasma Insulin (pmol·L^{-1})**	**Children**	55.16 ± 20.71	237.46 ± 36.64	142.76 ± 27.33	114.12 ± 28.76	243.32 ± 142.23	11179.95 ± 6887.40
	**Parents**	166.96 ± 61.30	274.81 ± 52.47	218.11 ± 38.00	153.14 ± 31.56	319.05 ± 207.42	9780.17 ± 8383.06
**Plasma Triglycerides (mmol·L^{-1})**	**Children**	1.14 ± 0.09	1.28 ± 0.14	1.25 ± 0.09	1.23 ± 0.09	1.44 ± 0.50	36.72 ± 48.95
	**Parents**	1.44 ± 0.11	1.44 ± 0.12	1.55 ± 0.11	1.51 ± 0.14	1.73 ± 0.46	18.81 ± 11.82

Table S1b. Blood glucose, plasma insulin and plasma triglyceride concentrations in children and parents across the rest trial. Data are mean ± SD.

		Rest					
**Variable**	**Group**	**Baseline**	**30-min postprandial**	**60-min postprandial**	**120-min postprandial**	**Peak Concentration**	**iAUC**
**Blood Glucose (mmol·L^{-1})**	**Children**	3.74 ± 0.62	5.26 ± 0.92	4.10 ± 0.48	4.57 ± 1.03	5.65 ± 0.71	100.10 ± 58.30
	**Parents**	4.38 ± 0.81	5.41 ± 0.87	4.39 ± 1.23	4.45 ± 0.78	5.53 ± 0.77	61.03 ± 34.38
**Plasma Insulin (pmol·L^{-1})**	**Children**	79.70 ± 30.73	246.48 ± 48.07	203.41 ± 44.43	119.53 ± 35.41	280.33 ± 190.88	11765.09 ± 7643.66
	**Parents**	200.83 ± 57.82	377.04 ± 65.61	209.47 ± 35.29	215.78 ± 34.10	400.52 ± 232.06	10947.94 ± 5430.23
**Plasma Triglycerides (mmol·L^{-1})**	**Children**	1.22 ± 0.11	1.22 ± 0.10	1.37 ± 0.13	1.44 ± 0.16	1.52 ± 0.59	21.52 ± 11.09
	**Parents**	1.28 ± 0.14	1.52 ± 0.17	1.61 ± 0.14	1.76 ± 0.15	1.86 ± 0.64	40.90 ± 32.72

Table S2. Inverse efficiency scores (ms) for the Stroop test, Sternberg paradigm and Flanker task in children and parents across the exercise and resting trial. Data are mean ± SD.

		Trial					
		Exercise			Rest		
**Variable**	**Group**	**Baseline**	**Immediately post**	**45-min post**	**Baseline**	**Immediately post**	**45-min post**
**Stroop Congruent (ms)**	**Children**	950 ± 26	924 ± 222	905 ± 228	954 ± 235	943 ± 244	932 ± 241
	**Parents**	889 ± 250	833 ± 248	818 ± 187	888 ± 204	892 ± 198	859 ± 189
**Stroop Incongruent (ms)**	**Children**	1363 ± 371	1339 ± 377	1340 ± 331	1392 ± 413	1317 ± 366	1270 ± 367
	**Parents**	1294 ± 428	1219 ± 375	1217 ± 355	1324 ± 363	1306 ± 385	1218 ± 342
**Sternberg One-item (ms)**	**Children**	694 ± 205	647 ± 171	677 ± 225	676 ± 164	681 ± 188	611 ± 152
	**Parents**	556 ± 156	543 ± 147	519 ± 115	604 ± 163	555 ± 143	533 ± 105
**Sternberg Three-item (ms)**	**Children**	854 ± 262	819 ± 249	932 ± 299	903 ± 247	843 ± 255	846 ± 248
	**Parents**	649 ± 149	664 ± 190	642 ± 153	720 ± 190	688 ± 161	641 ± 108
**Sternberg Five-item (ms)**	**Children**	1099 ± 347	950 ± 230	1227 ± 475	1103 ± 368	1010 ± 300	1057 ± 343
	**Parents**	796 ± 194	827 ± 206	787 ± 159	787 ± 161	830 ± 190	772 ± 134
**Flanker Congruent (ms)**	**Children**	779 ± 256	747 ± 322	726 ± 230	789 ± 266	778 ± 234	785 ± 294
	**Parents**	614 ± 174	615 ± 178	627 ± 179	643 ± 180	615 ± 165	589 ± 105
**Flanker Incongruent (ms)**	**Children**	1190 ± 1993	852 ± 459	878 ± 486	864 ± 283	827 ± 236	874 ± 347
	**Parents**	660 ± 177	672 ± 236	693 ± 229	692 ± 194	666 ± 193	631 ± 107

Table S3. Themes and sub-themes representing factors of families' enjoyment and the feasibility of implementing tag-rugby at home, with exemplar quotes.

Theme	Sub-theme	Quotes
**Perceived Enjoyment**	**Inclusive and enjoyable for the whole family**	"But they [daughters] tend to moan a lot, you know, like ohh you know, my legs are hurting. When are we there yet? So I think the tag-rugby thing brings the fun aspect to it where they don't actually realise that they're exercising. Yeah, because they're enjoying what they're doing, you know?" (Mother, low socioeconomic status)<br><br>"I think it was nice for me to do that with the children. Yeah, because I guess there are sports that the children do where they're doing it themselves and you're watching them, aren't you? So it's quite a nice activity to get involved in where, yeah, you know you have enjoyed and have fun together. Yeah. So, yeah I thought it was, It was nice" (Mother, low socioeconomic status)<br><br>"Yeah, I mean the weather was great and there were different tasks and it was interactive with ages and the families as well, so it was good" (Mother, middle socioeconomic status)<br><br>"I think it depends what it is [mode of physical activity], because if it was like that fun tag-rugby then that's fine. If it was at like a different level, then I'd rather do it myself, yeah" (Daughter, high socioeconomic status)

		"I think it was fun because we don't normally do stuff like that with each other, so it's like very different" (daughter, high socioeconomic status)<br><br>"Well they [parents] have longer legs and they can run faster so it gave us the opportunity to run as fast as possible and they can reach further so you have to be very good at getting through so it has got an element of surprise in it" (Son, high socioeconomic status)<br><br>"You just take all the contact out of it and then it's just running around and throwing a ball around. It's fun. Most people can kind of within reason catch and throw" (Father, high socioeconomic status)<br><br>"So I think well and also actually changing the rules a little bit helps because rugby is a bit weird in the sense that well, you can get into an argument about whether the ball's gotta go backwards because of the modern game. It doesn't go backwards. But anyway, by changing the rules so that they can just catch and throw the ball in any direction" (Father, high socioeconomic status)
	**Engaging elements that captivate both children and their parents**	"It was challenging because they're kids, you know, they're faster than that. So yeah, they've got more energy, though they're pushing us" (Mother, low socioeconomic status)<br><br>"I thought it was good because it was quite fast moving, and so it kept everyone interested. You were just, before you could get bored anything like that in terms of switching it kept everybody on their toes and kept everybody engaged so that was

		quite good" (Mother, middle socioeconomic status)<br><br>"It [tag-rugby game] almost got too, too free, but then some of us wanted to bring it back a little bit whereas the kids just wanted to carry on being silly. Not that it was a bad thing to be silly, we were just thinking more of the game" (mother, high socioeconomic status)<br><br>"I liked the drills except when it got repetitive so for quite a long time it didn't feel as fun" (Son, high socioeconomic status)<br><br>"It [tag-rugby game] was very very fun. It didn't feel that competitive, I felt like I was more having fun than competitive" (Son, high socioeconomic status)
**Feasibility of implementing family-based tag-rugby at home**	**Modality, intensity and duration of the session**	"No, it was ok because, because there was, there was a, there was half time, so there was half time. So, it's ok when doing it, she gave us a minute. Then we continue" (Mother, low socioeconomic status)<br><br>"Similar to how you might go out and walk the dog, you could be with your kids on the field playing something like that. Couldn't you, just to break the day up or something? You know, for half an hour or whatever" (Mother, low socioeconomic status)<br><br>"For me, I think it's nice to do it with multiple families. There's more interaction and the kids can

		have friends, and you know, we can make friends as well" (Mother, low socioeconomic status)<br><br>"I think it could've been a bit more high intensity just for myself because erm I didn't feel like I was going as fast as I could've but it was tiring" (Son, high socioeconomic status)<br>"You could make the pitch a bit bigger so that there was more intensity encouraged and more going on, I think that might help" (mother, middle SES)<br>"Yeah, especially in the summer when it's light and you can go out and enjoy the weather" (Father, high socioeconomic status)
	**Integrating tag-rugby into family life**	"It depends what world we're living in, if I could be doing something with him once a day I would, but in the reality we are living in with school, other extra curriculars we could only fit in once a week" (Mother, high socioeconomic status)<br><br>"Early morning or evening, because that leaves the rest of the day free, and then the amount of days available is bigger" (Father, middle socioeconomic status)<br><br>"I think because of school and work, probably weekends would be better, yeah" (Mother, middle socioeconomic status)<br><br>But yeah, it's just fitting it around with what they already do basically. I'd quite happily go and do it, you know, after school. Yeah, 45 minutes particularly in the summer, 45 minutes on an evening is nothing is it?" (Father, middle socioeconomic status)

		"Ermm mornings like after I wake up after breakfast. Erm, because I think morning is when I, I feel more energetic and it works better" (Son, middle socioeconomic status)
	**Traditional team sports and low impact sports as alternatives to tag-rugby**	"Because I quite like dodgeball and I quite like playing with my parents so that would be quite fun" (son, high socioeconomic status)<br>"So going for walks in the countryside would be my top one because they're really chilled out. And they [children] get on really well and they laugh a lot when we do go for walks. Yeah, I know they love going swimming into water parks. I really don't like it, so I wish I liked swimming more. Whenever you say, what do you want to do? They'll say family swim, which involves me going on slides and stuff. Erm yeah which I'm a bit funny about water, I don't really like the water. Erm, but yeah, that that would if I if I could get around it that would be a good one for us" (Mother, high socioeconomic status)<br><br>"And then especially if you can mix it in with going for pub lunch and taking, like, card games. So, that, that would be my favourite thing" (Mother, high socioeconomic status)<br><br>"Either dodgeball, football. But dodgeball, because it it is just fun. Then football, because I could skill them all" (Son, middle socioeconomic status)<br><br>"Football is the obvious one and then I would say I would like to play, and I'm not like a professional. I'm not like very good at it, but I'm OK. Badminton" (Son, middle socioeconomic status)

	"For me it would be basketball" (Son, high socioeconomic status)<br><br>"Yeah or netball or football" (Daughter, high socioeconomic status)
